# Detrusor overactivity assessment using ultrasound bladder vibrometry

**DOI:** 10.1088/1361-6579/ac2c5c

**Published:** 2021-10-29

**Authors:** David P Rosen, Douglas A Husmann, Lance A Mynderse, Troy F Kelly, Azra Alizad, Mostafa Fatemi

**Affiliations:** 1 Department of Physiology and Biomedical Engineering, Mayo Clinic College of Medicine & Science, Rochester, MN, United States of America; 2 Department of Urology, Mayo Clinic College of Medicine & Science, Rochester, MN, United States of America; 3 Department of Radiology, Mayo Clinic College of Medicine & Science, Rochester, MN, United States of America

**Keywords:** bladder, detrusor Overactivity, lamb waves, ultrasound elastography, urodynamics

## Abstract

*Objective*. Detrusor overactivity (DO) is a urodynamic observation characterized by fluctuations in detrusor pressure (*P*_det_) of the bladder. Although detecting DO is important for the management of bladder symptoms, the invasive nature of urodynamic studies (UDS) makes it a source of discomfort and morbidity for patients. Ultrasound bladder vibrometry (UBV) could provide a direct and noninvasive means of detecting DO, due to its sensitivity to changes in elasticity and load in the bladder wall. In this study, we investigated the feasibility and applying UBV toward detecting DO. *Approach*. UBV and urodynamic study (UDS) measurements were collected in 76 neurogenic bladder patients (23 with DO). Timestamped group velocity squared (${{{c}}_{{\mathrm{g}}}}^{2}$) data series were collected from UBV measurements. Concurrent *P*_det_ data series were identically analyzed for comparison and validation. A processing approach is developed to separate transient fluctuations in the data series from the larger trend of the data and a DO index is proposed for characterizing the transient peaks observed in the data. *Main Results.* Applying the DO index as a classifier for DO produced sensitivities and specificities of 0.70 and 0.75 for ${{{c}}_{{\mathrm{g}}}}^{2}$ data series and 0.70 and 0.83 for *P*_det_ data series respectively. *Significance*. It was found that DO can be feasibly detected from data series of timestamped UBV measurements. Collectively, these initial results are promising, and further refinement to the UBV measurement process is likely to improve and clarify its capabilities for noninvasive detection of DO.

## 1. Introduction

Detrusor overactivity (DO) is the urodynamic observation of involuntary contractions of the detrusor muscle of the bladder during the filling phase of urinary storage. To meet the criteria of DO the involuntary contractions must produce fluctuations in the detrusor pressure (*P*_det_) of larger than 15 cm of H_2_O over the baseline detrusor pressure as measured by urodynamic study (UDS) (Drake *et al* 2018, Rosier *et al* 2019). DO is common in neurogenic bladder patients (Goldmark *et al* 2014, Panicker *et al* 2015) and is important to diagnose in this population due to the risk of upper urinary tract issues (Liao 2015, Panicker *et al* 2015). In both neurogenic and non-neurogenic bladder patient populations, DO is also frequently accompanied by overactive bladder syndrome (OAB), which is characterized by symptoms of urinary urgency as well as frequency and nocturia (Abrams *et al* 2002). OAB is believed to occur in 10% of the population (Irwin *et al* 2011) and is associated with substantial reductions in quality-of-life (Sacco *et al* 2010).

Although assessment of DO in patients with OAB may be insightful for understanding the underlying etiology of said symptoms (Peyronnet *et al* 2019), clinical guidelines typically recommend assessing DO only when there is a substantial clinical justification. For instance, the American Urological Association guidelines recommend that DO only be assessed in non-neurogenic OAB after conservative treatments and drug therapies have failed (Winters *et al* 2012), a recommendation that is consistent with recent reviews such as (Aoki *et al* 2017, Al Mousa *et al* 2019). This is because of the potential for discomfort and morbidity associated with UDS, due to the catheterization of the urinary tract that is required for measuring bladder pressure. For instance, although rates of symptomatic urinary tract infections following UDS evaluations are highly variable and dependent on a variety of factors, a recent meta-analysis (Wu *et al* 2021) reported rates of symptomatic UTI across 6 studies of 23% (78/340) without administration of prophylactic antibiotics and 11% (48/418) if the test was performed with antibiotic prophylaxis.

Because of the potential complications of UDS, a variety of noninvasive techniques are being investigated for assessment of lower urinary tract symptoms such as OAB (Farag and Heesakkers, 2011, Gammie *et al* 2020). Ultrasound-based techniques, in particular, are promising as ultrasound is already a familiar tool in evaluation of lower urinary tract symptoms (Panicker *et al* 2015) and are also portable, safe, and cost-effective. Approaches to using ultrasound for assessment of DO have primarily focused on measuring geometric characteristics of the bladder. For instance, ultrasound has been used to measure bladder wall thickness, which has been proposed to correlate with DO due to detrusor hypertrophy (Bright *et al* 2010). Likewise, changes in bladder shape during contraction have been measured through ultrasound imaging (Glass Clark *et al* 2018, Gray *et al* 2019). However, the former has been shown to have limited diagnostic capabilities for DO (Rachaneni *et al* 2016, Latthe *et al* 2017), whereas the latter’s diagnostic potential has yet to be determined. Furthermore, the physical link between shape changes and DO is indirect and complex.

Recent work from our group has introduced ultrasound bladder vibrometry (UBV) as a new approach to assessing bladder elasticity through the measurement of induced Lamb waves along the wall of the bladder (Nenadic *et al* 2013, Fatemi and Nenadic 2016, Nenadic *et al* 2016, Bayat *et al* 2017). Because UBV is an elastographic technique, it has unique potential relative to other ultrasound-based methods, as it offers a more direct means of characterizing bladder elasticity and loading. Elastographic techniques have a well-documented sensitivity to the changes in tissue elasticity produced by tissue loading, including those produced by large deformations (Barr and Zhang 2012) and active muscle contraction (Gennisson *et al* 2005, Hug *et al* 2015). This sensitivity is increasingly leveraged as a potential means of assessing and tracking the fluid pressures within a variety of biological compartments, as such pressures induce mechanical tension within the walls of the biological compartment. Biological compartments where this association between pressure and elastographic measurement include the ventricles of the heart (Vejdani-Jahromi *et al* 2019), the diaphragm muscle and the lungs (Bachasson *et al* 2019), and large blood vessels (Wang *et al* 2019). For the bladder, previous work on UBV has shown medium-to-strong correlations of UBV measured Lamb wave group velocity squared (${{{c}}_{{\mathrm{g}}}}^{2}$) with UDS determined *P*_det_ (Bayat *et al* 2017).

Because UBV is sensitive to the elasticity and loading state of the bladder wall, we hypothesized that the temporal fluctuations in detrusor pressure can be usefully detected by UBV measurement. In this preliminary study, we investigated the feasibility of detecting DO by analysis of transient peaks in time-resolved series of UBV measurements on a cohort of neurogenic bladder patients undergoing routine UDS. To this end, we develop an approach to identifying fluctuations associated with DO in timestamped series of ${{{c}}_{{\mathrm{g}}}}^{2}$ measurements. We then propose an index that characterizes the intensity of such fluctuations which we compare statistically between DO and non-DO bladders.

## 2. Methods

In order to investigate the feasibility of detecting DO through analysis of UBV measurements, the following approach was used. ${{{c}}_{{\mathrm{g}}}}^{2}$ and *P*_det_ data series were gathered from experiments in which neurogenic bladder patients underwent concurrent UBV and UDS measurements. The collected ${{{c}}_{{\mathrm{g}}}}^{2}$ series were first preprocessed in order to remove statistically anomalous measurements and measurements with identified sources of variation unrelated to DO. Processing was then applied to the preprocessed data series in order to identify DO associated transient peaks. Finally, to characterize the decomposed series collected from a given experiment by a single number for statistical comparisons, a DO index, *I*, is proposed. A non-parametric statistical test and Receiver operator characteristic (ROC) curve analysis are then applied to measurement of *I* to evaluate the feasibility of detecting DO from UBV measurements. These steps are described in detail in the proceeding subsections.

### 2.1. Patient population

From April 2013 to January 2018, we evaluated 76 patient volunteers who were scheduled for a routine UDS were considered for the study. The study was composed of 76 adult patients with neurogenic bladder (16 females; 60 male). Inclusion criteria for the study were that patients be 21 years of age and have at least one of the following conditions: Neurogenic bladder, stress incontinence, benign prostatic hyperplasia, or voiding dysfunction. Patients were excluded in the case of a body mass index (BMI) greater than 35, a known neurologic disease other than those in the inclusion criteria, prolonged catheter drainage, previous radiation therapy, previous surgery in either the pelvis or bladder and pregnancy or breast-feeding. Patients’ ages ranged from 21 to 82 years, with the mean age of 50 ± 18 years. The BMI ranged from 17.3 to 34.2, with a mean BMI of 26.3 ± 4.60. Etiologies of neurogenic bladder symptoms included traumatic spinal cord injuries in 31% (23/76), multiple sclerosis in 31% (23/76), progressive neuropathy from an unknown etiology in 14% (11/76), spina bifida 11% (8/76), neural impingement from spinal column disc disease 9% (7/76), and Parkinson’s disease in 4% (3/75). The study was Health Insurance Portability and Accountability Act Compliant and was approved by the Mayo Clinic Institutional Review Board. A signed written informed consent with permission for publication was obtained from each enrolled patient prior to the study. Review of the urodynamic studies obtained within these volunteers revealed that 23 of the patients had documented DO during the performance of the UDS. All patients that consented to the study and completed both the UDS and UBV evaluations were included in the analysis.

### 2.2. UDS and UBV measurements

**Figure 1.**
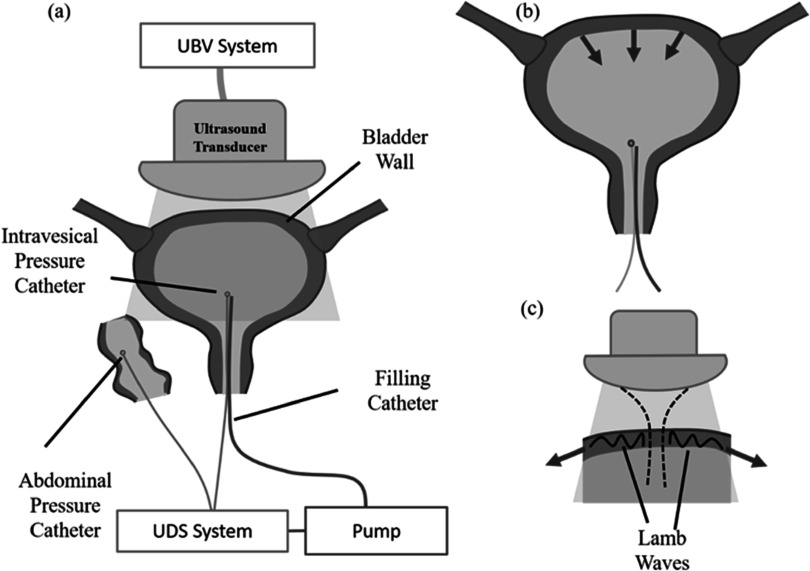
Diagrammatic representation of the basic elements of the UBV and UDS experiment setup. (a) UBV and UDS measurements were collected simultaneously during cystometric filling. (b) Contraction of the detrusor muscle induces fluctuations in pressure within the bladder resulting in fluctuations in *P*_det_. (c) Detrusor contraction also produce tensile loading in the bladder wall. We hypothesis that this change in the loading state of the bladder wall will result in fluctuations in the Lamb wave velocities measured by UBV.

UBV measurements were collected during the patient’s routine cystometry and UDS. Regarding the urodynamic techniques we used standardized techniques as outlined by the International Continence Society at a filling rate of 25 ml min^−1^ in either a supine or sitting position (Rosier *et al* 2017). It is noteworthy that prior studies in our laboratory revealed no difference in UBV measurements between the supine and sitting positions (Adusei *et al* 2021). During the filling phase of the UDS, filling was stopped every 50 ml up to the maximum patient capacity. The pressure sustained by the detrusor muscle of the bladder (i.e. *P*_det_) was recorded in a continuous fashion throughout bladder filling with a dense sampling rate (≥2 Hz) that is in line with the International Continence Society’s standards for urodynamic equipment (Gammie *et al* 2014). DO on the UDS was defined by standard urodynamic criteria (Drake *et al* 2018, Rosier *et al* 2019). UBV measurements were obtained episodically during the time of bladder filling with 2–3 acquisitions performed at each volume. At each stoppage volume, the probe was placed on the lower abdomen targeting the anterior bladder wall to collect measurements and was removed during filling to the next volume. At the time of acquisition of the UBV measurement, a concurrent *P*_det_ measurement was marked on the UDS chart that was used for DO diagnosis and timestamps were recorded for both measurements. It is noteworthy that the UBV measurements and marked *P*_det_ measurements are episodic and will be substantially undersampled compared the near continuously sampled urodynamic evaluations. The resulting series of *P*_det_ measurements correspond to the *P*_det_ channel from the UDS chart but undersampled identically to the UBV measurements. Figure 1 illustrates the experimental set-up during these measurements.

**Figure 2.**
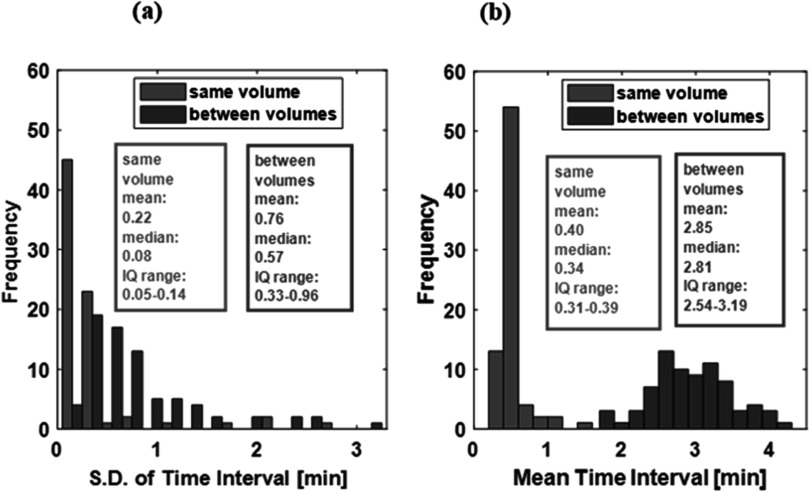
Histograms of sampling interval statistics for each bladder experiment. The standard deviations (S.D.) (a) and means (b) of sampling intervals of each experiment were calculated for intervals between measurements collected at the same filling volume (orange) and intervals between measurements collected before and after the transition to a different filling volume (blue). The statistics reported in the text box are for the respective histogram (IQ range: interquartile range).

Figure 2 shows histograms of the means and standard deviations of time intervals between the episodic UBV measurements for each experiment. The time interval statistics are separated into time intervals between measurements collected at the same stoppage volume and between stoppage volumes. Separate means and standard deviations are collected for measurements taken at the same filling volume and measurement taken between filling volumes.

The UBV system and measurement sequence has been previously reported in Bayat *et al* (2017) and Nenadic *et al* (2016). UBV measurements were collected using a programmable ultrasound system (Verasonics, Redmond, WA), and a curvilinear array transducer (C4-2, ATL/Philips, Bothewell, WA) with a center frequency of 2.5 MHz. A single 600–900 *μ*s tone burst acoustic pulse (2.5 MHz driving frequency) was applied to the wall of the bladder to induce a broad spectrum of waves in the tissue through the mechanism of acoustic radiation force. An ultrafast plane wave imaging sequence then captured the resulting Lamb wave propagation along the bladder wall. Ultrasonic plane waves were transmitted at a pulse repetition frequency of 7500 Hz and 3 steering angles (−4°, 0°, 4°) were used for coherent compounding of the ultrafast images (effective frame rate 2500 Hz). The transmit frequency and pulse duration of the imaging pulses were 3 MHz and 0.33 *μ*s respectively. Coherent compounding was used to limit the effect of sidelobes that is known to distort the displacement tracking in heterogenous tissues. See Montaldo *et al* (2009) for a description of the benefits of coherent compounding in the tracking of transverse waves for elastography. Particle motion velocity was estimated from the resulting in-phase and quadrature (IQ) data using an autocorrelation technique (Loupas *et al* 1995) and an ensemble length of 2 pulses. The group velocity (*c*_g_) of the Lamb wave propagating along the wall of the bladder was then estimated as it was in Bayat *et al* (2017): by collecting the time-to-peak of the particle velocity wave profile for each lateral location along the bladder wall and fitting a regression line to estimate the slope of the arrival times relative to position. Because elasticity is expected to correlate with the square of the wave speed, as is more rigorously the case with bulk waves, the group velocity was squared (i.e. ${{{c}}_{{\mathrm{g}}}}^{2}$) for analysis, as was also done in the previous paper (Bayat *et al* 2017).

### 2.3. Data preparation and peak identification

Here the peak identification process is described, wherein the ${{{c}}_{{\mathrm{g}}}}^{2}$ or *P*_det_ data series collected from the concurrent UBV/UDS experiments are preprocessed and decomposed into two series: one containing transient peaks identified by the processing and hypothesized to correspond to DO, and another containing effectively the lower envelope (LE) that tracks the larger time trend of the data series during bladder filling. All processing and subsequent statistical analysis of ${{{c}}_{{\mathrm{g}}}}^{2}$ and *P*_det_ data series were applied using MATLAB 2019a (Mathworks Inc., Natick, MA).

All of the same preprocessing and processing applied to ${{{c}}_{{\mathrm{g}}}}^{2}$ measurement series for peak identification and subsequent statistical analysis was also applied to concurrently collected *P*_det_ measurement series (i.e. *P*_det_ measurements marked on the UDS at the timepoints of UBV acquisitions). This was done based on the hypothesis that detrusor contractions due to overactivity produce similar responses in ${{{c}}_{{\mathrm{g}}}}^{2}$ and *P*_det_ measurements (this hypothesis is illustrated in figures 1(b) and (c)). As such, applying the same analysis to concurrent *P*_det_ measurements as that applied to ${{{c}}_{{\mathrm{g}}}}^{2}$ measurements would be expected to produce similar statistical discrimination of DO. It also provides validation of the proposed analysis, as the concurrent *P*_det_ measurements are undersampled as compared to the full UDS chart used by the urologist to diagnose DO.

**Figure 3.**
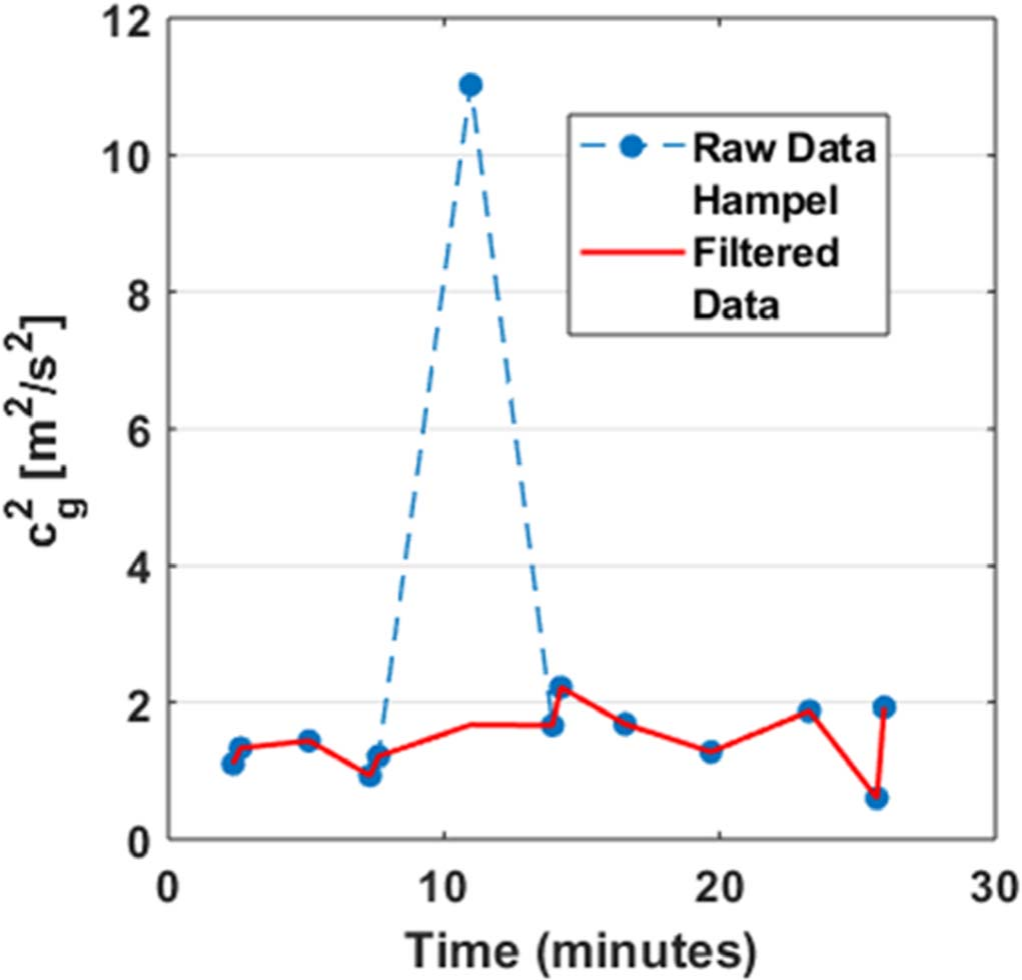
Example data series before Hampel filtering (blue) and after Hampel filtering (red). Note that the data series is otherwise unchanged except for the replacement of a single large outlier.

Prior to the peak identification process, several pre-processing steps were applied to the ${{{c}}_{{\mathrm{g}}}}^{2}$ and *P*_det_ data series. These were done in order to avoid fluctuations in the series that are expected to be unassociated with DO. Values of the given data series which were marked at the time of the experiment as having occurred during a bladder leak were removed from the series. Measurements collected at the largest fill volume of each experiment were also removed to avoid the inclusion of normal detrusor contraction associated with the onset of voiding (Wyndaele *et al* 2011). Occasionally, a severe outlier measurement would be present in either the ${{{c}}_{{\mathrm{g}}}}^{2}$ or *P*_det_ data series, as illustrated in figure 3. Such outliers could be due to, for instance, patient motion during measurement or unusually weak UBV signals. These outliers were handled by applying a statistical outlier filter to all of the data series (regardless of whether or not an outlier measurement was suspected). The statistical filter used was a Hampel filter (Pearson *et al* 2016) with an 11-point window width and an outlier cut-off of 6 standard deviations. Details of the implementation of this outlier filter are provided in appendix A.

**Figure 4.**
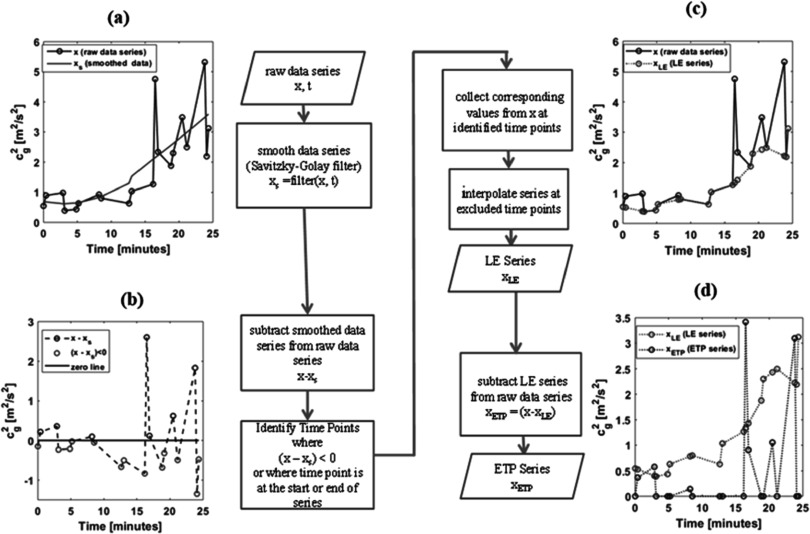
Flow chart of peak identification process. (a) Raw data series is smoothed using a 2nd-order Savitzky–Golay filter. (b) The Raw data is differenced by the smoothed data and the time-points where values falling below the smoothed curve (i.e. having a negative value) are identified. The identified time points are used to define the LE series from the raw data series by interpolating points in between (c). (d) The difference between the LE series and the raw data series determines the ETP series.

Because the ${{{c}}_{{\mathrm{g}}}}^{2}$ and *P*_det_ data series often showed large time-varying trend due to bladder filling, accompanied by smaller fluctuations due to either DO-associated contractions or measurement noise, DO associated peak identification required some means of separating these fluctuations from the larger time-varying trend. The approach we propose for making this separation is illustrated in figure 4 and is analogous to detrending of a time series before analysis (Casella *et al* 2008). However, rather than assuming some common functional form of the large time-varying trend, we collect points that constitute the lower portion of the trend by first smoothing the data (See solid red curve in figure 4(a)) through a 2nd-order Savitzky–Golay (Savitzky and Golay, 1964) filter with a smoothing factor of 0.95. The points falling below the smoothed series are then collected, as shown in figure 4(b). After interpolation the time-points between these lower points, the resulting series is what we will hereafter refer to as the LE series. Figure 4(c) shows the LE series plotted over the original series. It can be seen that by subtracting the LE series from the original series, a new series that captures transient peaks observed on top of the larger trend of the measurement series is collected. This series is shown in figure 4(d) in blue and will be referred to as the ensemble of transient peaks (ETP) series. A detailed description of the processing steps involved in this method is provided in appendix A.

### 2.4. DO index

In order to make statistical and diagnostic comparisons between the peak identification of DO and non-DO bladders, we proposed a DO index that characterizes the increased signal energy associated with fluctuations due to DO. To be applied to these current experiments, such a DO index should be, in principle, comparable between experiments of variable durations as different patients will have different maximum capacities. Likewise, it should also be comparable between experiments with varied ranges in UBV measurement. For this purpose, we defined the proposed DO index, denoted as *I*, as follows:

\begin{eqnarray*}I=\displaystyle \frac{{\mathrm{RMS}}\left({{x}}_{{\mathrm{ETP}}}\right)}{{\mathrm{RMS}}\left({{x}}_{{\mathrm{LE}}}\right)}\approx \displaystyle \frac{\sqrt{\,\displaystyle \frac{1}{{{T}}_{2}-{{T}}_{1}}\displaystyle {\int }_{{{T}}_{1}}^{{{T}}_{2}}{{x}}_{{\mathrm{ETP}}}{\left({t}\right)}^{2}}}{\sqrt{\,\displaystyle \frac{1}{{{T}}_{2}-{{T}}_{1}}\displaystyle {\int }_{{{T}}_{1}}^{{{T}}_{2}}{{x}}_{{\mathrm{LE}}}{\left({t}\right)}^{2}}}=\displaystyle \frac{\sqrt{{{E}}_{{\mathrm{ETP}}}}}{\sqrt{{{E}}_{{\mathrm{LE}}}}}.\end{eqnarray*}

Here ${{x}}_{{\mathrm{ETP}}}$ denotes the ETP data series and ${{x}}_{{\mathrm{LE}}}$ the LE data series, for a given experiment. Likewise, ${{x}}_{{\mathrm{ETP}}}(t)$ and ${{x}}_{{\mathrm{LE}}}(t)$ correspond to their continuous time counterparts. Essentially, $I$ is an approximation of the ratio of root signal energies (denoted as $\sqrt{{{E}}_{{\mathrm{ETP}}}}$ and $\sqrt{{{E}}_{{\mathrm{LE}}}}$ respectively) from the transient peaks and the LE. As such, it is expected that data series from bladders with DO will have a larger signal energy in ${{x}}_{{\mathrm{ETP}}}(t)$ relative to ${{x}}_{{\mathrm{LE}}}({t}),$ and thus a larger $I,$ than bladders without DO. Additionally, normalizing $\sqrt{{{E}}_{{\mathrm{ETP}}}}$ by $\sqrt{{{E}}_{{\mathrm{LE}}}}$ should mitigate for differences between bladders in terms of the response of their ${{{c}}_{{\mathrm{g}}}}^{2}$ series to filling and DO contraction. Such differences between bladders could be due to differences in bladder wall thickness and passive elasticity. Finally, because $I$ is unitless, it is reasonable to make comparisons between experiments, as longer experimental durations and wider ranges in measured ${{{c}}_{{\mathrm{g}}}}^{2}$ or *P*_det_ are accounted for by normalizing ${\mathrm{RMS}}({{x}}_{{\mathrm{ETP}}})$ by ${\mathrm{RMS}}\left({{x}}_{{\mathrm{LE}}}\right)$ as is done in equation (1).

### 2.5. Statistical analysis

Statistically significant differences in the DO index between DO and non-DO bladders was assessed using a non-parametric hypothesis test, i.e. the Wilcoxon Test (Mann and Whitney, 1947). ROC analysis was also applied as a preliminary evaluation of diagnostic potential. Optimal cut-offs were estimated for the DO index using closest-to-(0,1) criterion (Perkins and Schisterman 2006) (i.e. the point on the curve nearest to the upper left corner of the ROC plot). We also investigated the potential correlation between the DO index and the patients age and bladder compliance measured from the UDS as they could be covariant with the DO index. The Spearman’s correlation coefficient was calculated for DO for all bladders. The bladder compliance was defined as in Wyndaele *et al* (2011) and had units of ml/cmH_2_O.

## 3. Results

**Figure 5.**
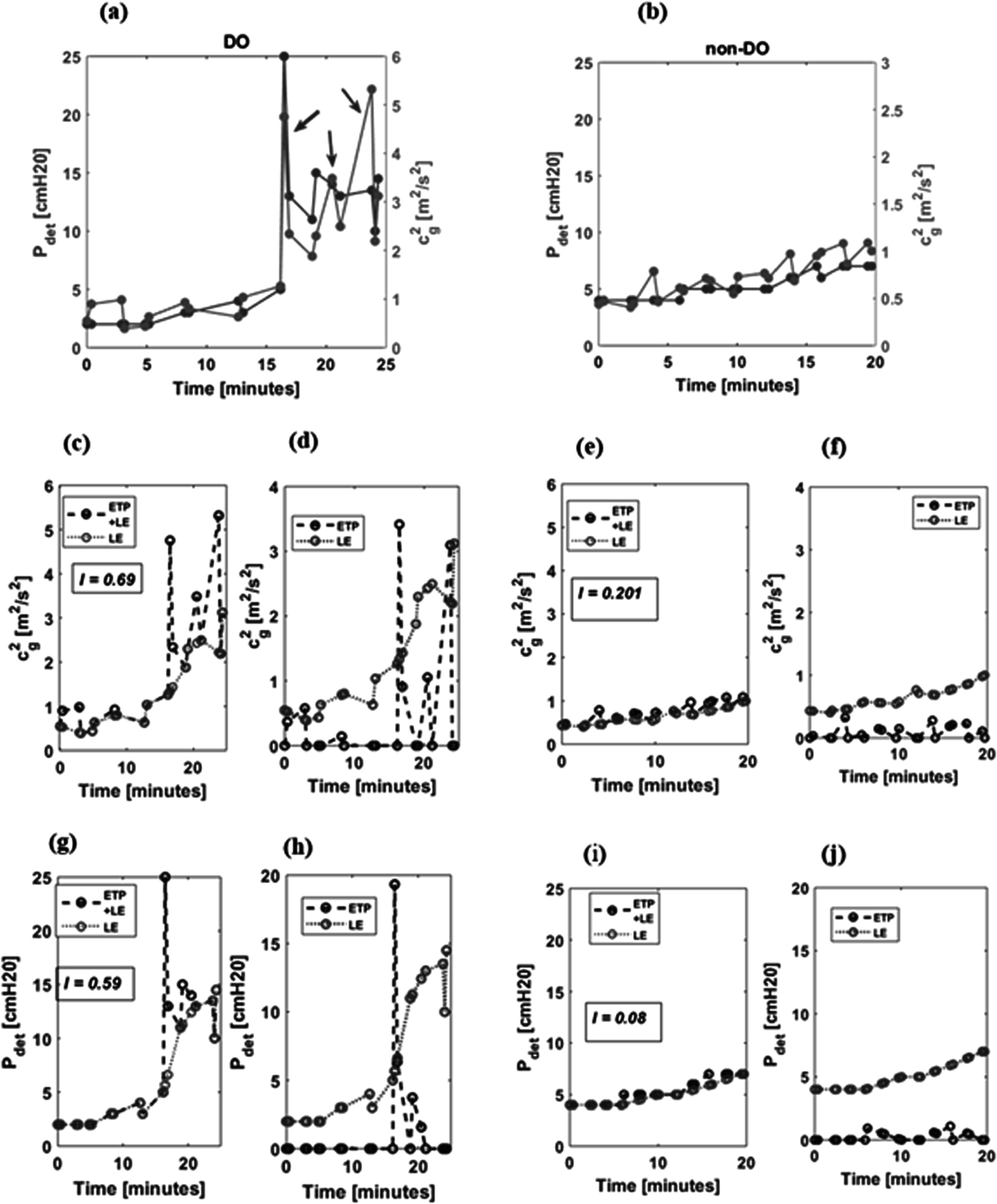
Representative ${{{c}}_{{\mathrm{g}}}}^{2}$ and *P*_det_ data series for DO (left) and non-DO (right) bladders. Top row displays dual-axis plots of both data series, *P*_det_ and ${{{c}}_{{\mathrm{g}}}}^{2}.$ The red arrows in (a) indicate the peaks associated with DO. The same series are shown in the middle (${{{c}}_{{\mathrm{g}}}}^{2}$) and bottom (*P*_det_) rows are shown with lower envelope (red) overlaid on the raw series (blue) (c), (e), (g), (i) to illustrate the decomposition of the raw series into ETP and LE series. The separated ETP (blue) and LE (red) series used to calculate the signal index (*I*) are shown in (d), (f), (h), (j). Note that the identified ensemble of peaks for the data series is the difference between the raw series and the lower envelope.

Results are presented by first demonstrating the proposed characterization and associated observations on example UBV and UDS measurements from a bladder with DO (figure 5(a)) and a bladder without DO (figure 5(b)). The characterization is then verified in the remaining bladders and evaluated for its diagnostic potential by statistical analysis of the proposed DO index.

Figure 5 presents example analysis of ${{{c}}_{{\mathrm{g}}}}^{2}$ and *P*_det_ data series for the DO and non-DO bladders collected over the course of the UDS study. Note that for the ${{{c}}_{{\mathrm{g}}}}^{2}$ and *P*_det_ data series from the example bladder with DO, several transient peaks are observed (indicated by red arrows in figure 5(a)). Furthermore, these peaks appear to be separable from the larger trend of the data series when decomposing the series into ETP and LE signals respectively (see red and blue curves in figures 5(d), (h)). Although transient fluctuations are observable in the non-DO bladder, their associated signal energy is comparatively low. This pattern is captured in our proposed DO index, *I*. In particular, a higher value of *I* is observed in the example DO bladder for both ${{{c}}_{{\mathrm{g}}}}^{2}$ (*I* = 0.69; See figures 5(c), (d)) and *P*_det_ (*I* = 0.59; See figures 5(g), (h)) than in the example non-DO bladder (*I* = 0.20 for ${{{c}}_{{\mathrm{g}}}}^{2}$ and *I* = 0.08 for *P*_det_; See figures 5(e), (f) and 1(i), (j) respectively).

**Figure 6.**
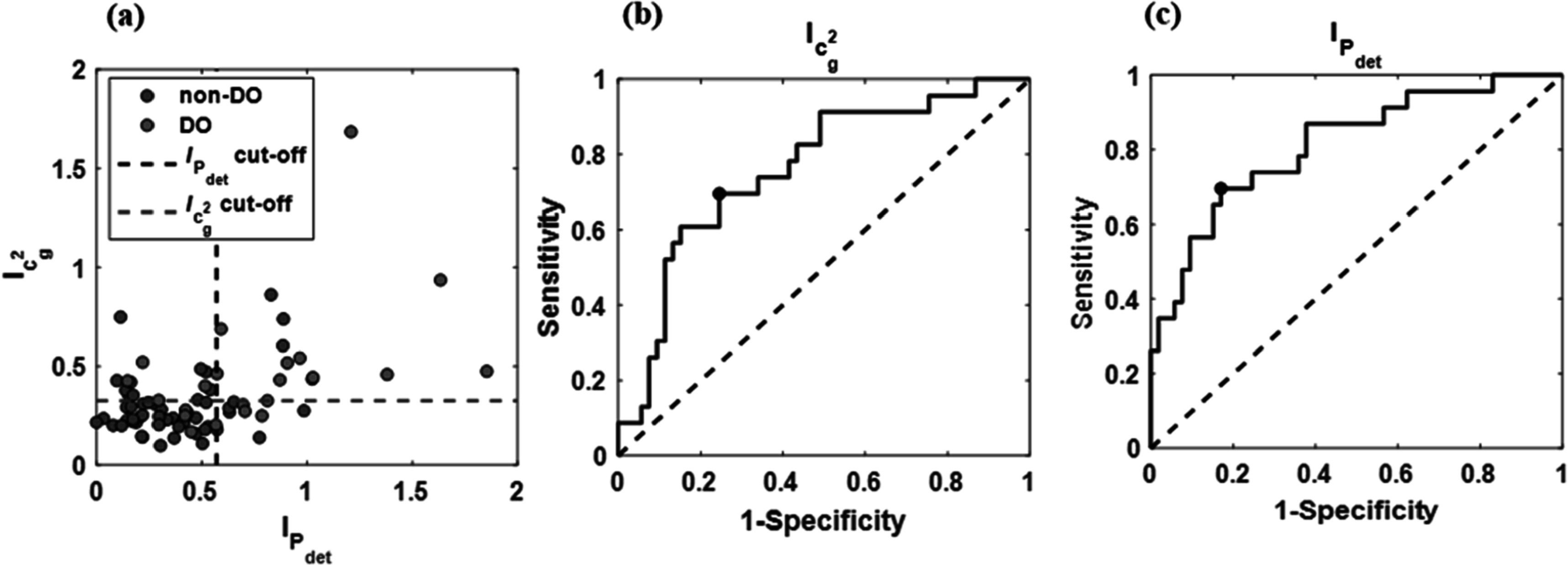
Graphical results of statistical and diagnostic analysis. Scatter plot of ${{{c}}_{{\mathrm{g}}}}^{2}$ signal index against *P*_det_ signal index (a). The black and red dashed lines denote ${{{c}}_{{\mathrm{g}}}}^{2}$ and *P*_det_ cut-offs respectively. ROC curves producing by using the signal index (*I*) as a classifier for DO applied to ${{{c}}_{{\mathrm{g}}}}^{2}$ (b) and *P*_det_ (c). The blue circular markers denote the selected optimal cut-off.

Figure 6 graphically presents these statistical results through a scatter plot of DO indices calculated for UBV/UDS experiments from all 76 patients (figure 6(a)) as well as accompanied ROC curves for ${{{c}}_{{\mathrm{g}}}}^{2}$ and *P*_det_ (figures 6(b), (c)). In agreement with the example bladder shown in figure 5, median DO indices for ${{{c}}_{{\mathrm{g}}}}^{2}$ and *P*_det_ were larger in bladders with DO (0.43 and 0.70 respectively) than they were in bladders without DO (0.25 and 0.31). The Wilcoxon test suggests statistically significant differences between DO and non-DO bladders for *I* calculated from ${{{c}}_{{\mathrm{g}}}}^{2}$ (*p* < 0.01) and from *P*_det_ (*p* < 0.01). Figures 6(b), (c) present the associated ROC curves of *I* calculated for ${{{c}}_{{\mathrm{g}}}}^{2}$ and *P*_det_ respectively. The corresponding cut-off values were 0.327 for *I* calculated for ${{{c}}_{{\mathrm{g}}}}^{2}$ and 0.567 for *I* calculated from *P*_det_. Associated diagnostic performance metrics for these cut-offs, including sensitivities and specificities, are reported in table 1.

**Table 1.** Diagnostic performance metrics of the DO Index.

	${{{c}}_{{\mathrm{g}}}}^{2}$	*P*_det_
AUC	0.76	0.81
Optimal cut-off	>0.327	>0.567
Sensitivity	0.70	0.70
Specificity	0.75	0.83
Accuracy	0.72	0.78

Although classification characteristics of peak characterization were comparable between ${{{c}}_{{\mathrm{g}}}}^{2}$ and *P*_det_ (i.e. comparable sensitivity, specificity, and AUC; See table 1), from figure 6(a), it can be seen that a portion of their respective classifications was in disagreement for a minority of the data (i.e. upper left and lower right quadrants marked by the respective cut-off lines). This disagreement corresponded to 31.6% of the bladders (8/23 DO bladders and 16/53 non-DO bladders).

Age and bladder compliance had nonsignificant Spearman’s correlation coefficients for the DO index calculated on ${{{c}}_{{\mathrm{g}}}}^{2}$ (*ρ* = −0.134 and *p*-value 0.25 for Age and DO-index and *ρ* = −0.23 and *p*-value 0.051 for bladder compliance). This was similarly the case for Spearman’s correlations with the DO index calculated from *P*_det_ (*ρ* = −0.023 and *p*-value 0.83 for Age and DO-index and *ρ* = −0.12 and *p*-value 0.31 for bladder compliance).

## 4. Discussion

In this study, we investigated the feasibility of detecting DO through analysis of transient peaks in UBV measurements. We characterized peaks in these measurements through a proposed DO index that characterizes the differing signal energies between peaks and LE. Our results show a statistically significant difference in *I* calculated for ${{{c}}_{{\mathrm{g}}}}^{2}$ between DO and non-DO bladders. ROC analysis showed this analysis of ${{{c}}_{{\mathrm{g}}}}^{2}$ series produced a sensitivity of 0.70 and a specificity of 0.75 for detecting DO. This is comparable to the sensitivity and specificity produced by applying the same analysis to the concurrent *P*_det_ measurements recorded from the UDS (0.70 and 0.83, respectively). Recall that the concurrent *P*_det_ measurement series is effectively the *P*_det_ channel from the UDS chart but with undersampling that matches ${{{c}}_{{\mathrm{g}}}}^{2}$ and that the analysis was applied to it for validation of the processing approach as well as comparison. Therefore, this comparability suggests promising potential for UBV in detecting DO.

Although the differences in DO classification between ${{{c}}_{{\mathrm{g}}}}^{2}$ and *P*_det_ observed in figure 6(a) may be due to random variations, it may also be a consequence of the localized nature of the UBV measurement. Because UBV measures wave propagation along a segment of the bladder wall, it likely only captures the contractile activity of the tissue along the measured segment. In contrast, transient fluctuations of *P*_det_ are a function of the net contractile activity of the detrusor muscle over the full bladder. Currently, there is some preclinical evidence for spatiotemporal variation of detrusor contraction over the bladder wall (Lentle *et al* 2015), where bladder contractions appear to propagate spatially along the bladder wall. This might explain why the ROC characteristics for *I* were similar when calculated from ${{{c}}_{{\mathrm{g}}}}^{2}$ versus *P*_det_, while showing differing classifications in 31.6% of the bladders. The development of UBV to allow for spatially resolved measurements along the bladder wall would offer the possibility of investigating this hypothesis *in vivo*.

The UBV measurements in our study were conducted concurrently with UDS measurements at increasing filling volumes. The advantage of this approach is that the UBV and concurrent UDS measurements were collected at the same time as the UDS charts used for urologist diagnosis of DO (ground truth), which removes the variability that would have been introduced if the UDS examination used to confirm the presence of DO were performed at different times. Another benefit of this approach is that we were able to compare our peak analysis to both ${{{c}}_{{\mathrm{g}}}}^{2}$ and *P*_det_ that were acquired at the same time. Our results showed that applying our analysis to *P*_det_ from UDS data and ${{{c}}_{{\mathrm{g}}}}^{2}$ from UBV data provide similar diagnostic performance. This suggests that UBV and UDS provided similar information relative to the proposed diagnostic index. This supports the potential of UBV as a noninvasive alternative to UDS for DO diagnosis.

The benefits of our approach came at the price of some limitations. One such limitation is the study population which included only patients with neurogenic DO. Although patients with idiopathic DO were not investigated here, we expect the UBV response to be the same as observed here for neurogenic DO. The only clinical distinction between idiopathic DO and neurogenic DO is that the latter has no known underlying pathology associated with it. However, the urodynamic observation is the same. Because UBV and UDS responses are driven by the same underlying mechanisms (i.e. changes in bladder wall elasticity and loading), idiopathic DO should produce the same UBV response as neurogenic DO.

Another limitation is that the transducer was episodically removed from the abdomen during the experiment. It can be expected that, because of changes in the probe position as well as the changes in the bladder volume, the ultrasound beam may not target exactly the same location of the bladder for each measurement. However, such errors can be acceptable assuming relatively uniform elasticity and loading along the anterior bladder wall.

A more substantial limitation for this study is that the sampling intervals between UBV measurements were relatively large, especially between filling volumes (figure 2(b)). Furthermore, because UBV measurements were collected episodically at the discrete stoppage filling volumes, they were irregularly sampled in time and there was variation in the time intervals between measurements (figure 2(a)). Although the results of this study indicate that the components of the processing and preprocessing appear to be robust to such a limitation on average, it is likely that many of the false positives and false negatives observed in this investigation are due to this limitation. For instance, with sparse sampling points and a bladder with infrequent detrusor contractions, the outlier filter may classify a measurement collected at the peak of a contraction as an anomalous outlier. Likewise, the Savitzky–Golay filter used to define the LE of the data series may be overly influenced by a few measurements of large values when sampling is limited, though this is less likely given the effect of the outlier filter that is applied before it. A related source of error is that transient peaks in the full UDS may be missed due to unusually large delays between UBV measurements.

The limitations that accompany the large sampling intervals are acceptable for the purposes of this current study, which was to prove the concept of noninvasive detection of DO through UBV measurement. However, precise characterization of the amplitudes and durations of DO contractions through their associated UBV response will require denser time sampling than was collected here. Refinements to the UBV experimental setup that include dense and regular temporal sampling could potentially produce greater diagnostic strength. Furthermore, such refinements would permit time series and frequency-domain based analysis to be applied to the UBV measurement series, such as was adopted in a study by Cullingsworth *et al* (2018). It should be noted that the peak identification process and DO index proposed in this investigation could be readily applied to measurements with dense and regular temporal sampling with minor adaption and that such improvements would reduce the occurrence of false positives and false negatives due to the effects of sparse sampling on the filters used in the current processing. With all of this in mind, these current results should be considered as a first step in applying UBV toward the diagnosis of DO. Future studies are planned to implement such improvements to our experimental approach to UBV.

Keeping in mind the advantages and limitations of this initial investigation and the need for future refinements to UBV targeted toward assessment of DO, these initial results are promising and motivating for future investigation. While UBV was coupled with UDS in this study for verification purposes, we envision that practical application of UBV toward DO diagnosis would be completely non-invasive. In such a case, the cystometric filling used in these current experiments would be replaced by the natural filling of the bladder after oral hydration as has been done for UBV in Nenadic *et al* (2016). It should be noted that the application of non-invasive UBV still need to be monitored by a clinician in order to position the probe and evaluate factors affecting the measurement. Many factors to effectively applying such a procedure are yet to be determined, including the ideal sampling duration and rate and the preferred filling volume for the detection of DO. Additionally, it should be noted that UDS provides more information to urologists than assessment of DO, such as bladder compliance. Though further development of UBV may provide non-invasive assessment for many of these components due to its correlation with *P*_det_ (Bayat *et al* 2017), measurements such as urinary flow rate would require added techniques beyond UBV in order to replace all or most of the its diagnostic applications. Therefore, as UBV’s scope of application is explored relative to lower urinary tract symptoms, it is likely that the design of practical UBV procedures will develop to incorporate such applications.

The potential benefit of UBV over UDS toward assessment of DO is the possibility of sensing DO without the need for catheterization. This would allow for its use in DO diagnosis when UDS cannot be justified due to the risks of morbidity and discomfort (Aoki *et al* 2017, Al Mousa *et al* 2019). Catheterization may also be a source of irritation to the bladder wall. As noted in Abrams (2003), such irritation could explain the high rates of DO (up to 60%) observed in ambulatory UDS (Brown and Hilton 1997). UBV could offer a means of testing this hypothesis as well as offer an alternative assessment of DO that is closer to everyday living (Abrams *et al* 2002). Because ultrasound is portable, and potentially even wearable (Pashaei *et al* 2020), UBV may offer an attractive avenue for assessing DO during ambulation which would substantially increase the urologist’s acumen. We envision future investigations of UBV-based detection of DO performed without the use of concurrent UDS and on a larger patient population to investigate its utility under a greater variety of conditions and sources of DO.

## 5. Conclusion

This study investigated the feasibility of detecting DO through UBV, which is a noninvasive ultrasound technique. We found that DO could be detected from UBV measurements using the proposed peak identification processing and DO index. Although preliminary, these initial results suggest that UBV has potential as a noninvasive tool for detecting DO.

## Conflict of interest disclosure

Mostafa Fatemi is an inventor of US patent 9,345,448 on the technology used in this study. Mostafa Fatemi and the other authors declare no relationship with any company whose products or services may be related to the subject matter of the article. The authors also affirm that they do not have any potential financial interest related to the technology used in this study.

## Appendix A. Description of the statistical filter used for outlier removal

In order to remove outliers, a statistical outlier filter called a Hampel filter (Pearson *et al* 2016) was applied to the ${{{c}}_{{\mathrm{g}}}}^{2}$ and *P*_det_ data series. This filter works by replacing points determined to be outliers with the median of the sample window. The filter uses the following criteria for identifying outliers: a given sample from the data series, *x*_i_, which is the center point of a sliding window with a median of *m*_i_, is considered an outlier if

\begin{eqnarray*}\left|{{x}}_{{i}}-{{m}}_{{i}}\right|> {{n}}_{{\mathrm{\sigma }}}{{\mathrm{\sigma }}}_{{i}}.\end{eqnarray*}

Here, ${{n}}_{{\mathrm{\sigma }}}$ is a threshold number of standard deviations and ${{\mathrm{\sigma }}}_{{i}}$ is the estimated standard deviation of the sampling window. The standard deviation of the sampling window is estimated from the median absolute deviation as

\begin{eqnarray*}{\sigma }_{{i}}={k}\,\mathrm{median}\,(\left|{{x}}_{{i}-{w}}-{{m}}_{{i}}\right|,\,\ldots ,\,\left|{{x}}_{{i}+{w}}-{{m}}_{{i}}\right|),\end{eqnarray*}

where ${w}$ is the number of pixels on either side of the centerpoint of the window and the parameter,$k,$ scales the median absolute deviation to the standard deviation. For a standard normal distribution $k\approx 1.483$ (Hampel 1974, Rousseeuw and Croux 1993). For analysis of the UBV data, ${{n}}_{{\mathrm{\sigma }}}$ was set to 6 and ${w}$ was set to 5 for the following reasons. Although the filter does not permit a fixed time window for unevenly sampled data, it does not modify the signal except where an outlier is detected. Likewise, the window is set to be large relative to the length of the signal (11 points within the window for ${w}=5$ versus ~16 points based on the median sampling interval and median experimental duration.) By setting the outlier threshold conservatively in terms of the number of anomalous outliers it is likely to identify, the filter alters only extreme outlying points in the series and leaves the remaining points unaltered (as illustrated in figure 3). Because few points are modified by the filter and the sample window is large, the effect of unfixed time window is expected to be minimal. Setting ${{n}}_{{\mathrm{\sigma }}}$ to 6 means that, for a Gaussian distribution, 99.9997% of the data is left unaltered. While we do not speculate that data within the filter window should follow a Gaussian distribution *a prior*, the filter offers a reliable and objective heuristic for identifying anomalous outliers within the data series that does not rely on human judgment.

## Appendix B. Detailed description of ETP and LE series decomposition

The following is a detailed description of the processing steps used to decompose data series of UBV and UDS measurements into ETP and LE series. These steps are illustrated in figure 4.

(1) A smoothing filter was applied to the raw data series (*x*) to capture any trend in the data series associated with bladder filling (figure 4(a)) Smoothing is applied to the series through a 2nd-order Savitzky–Golay filter (Savitzky and Golay, 1964) through MATLAB’s ‘smooth’ function. The Savitzky–Golay filter applies a (2nd-order) polynomial regression to data within a sliding window. Because the time intervals are irregularly spaced (figures 2(a), (b)), a smoothing factor is used in place of a discrete window size. The smoothing factor takes on a value between 0 and 1, which corresponds to the percent of signal energy being attenuated by smoothing (e.g. a smoothing factor of 0.5 would attenuate 50% of the signal energy). The smoothing factor was set to 0.95 in order to filter all fluctuations in the data series except for the main trend of the data.
(2) After smoothing, *x* is differenced with the filtered data series (*x*_s_) and time-points associated with negative values of the differenced series are identified (i.e. time points were (*x*−*x*_s_) < 0).The start and end time-points were also included in the identified time points, so that increases at the time boundary are not treated as transient peaks. (See figure 4(b) for illustration).
(3) Values from *x* at the identified time-points are then collected. Linear interpolation is then used to assign values at the time-points that were not identified in step two. The resulting series has the same number of elements as *x*. We refer to the resulting series as the LE series (*x*_LE_). (See figure 4(c) for illustration).
(4) The ETP series (*x*_ETP_) is then determined by differencing the *x*_LE_ from *x*. (See figure 4(d) for illustration).

The ETP and LE series can then be used for further analysis.
